# miR-103 Regulates Oxidative Stress by Targeting the BCL2/Adenovirus E1B 19 kDa Interacting Protein 3 in HUVECs

**DOI:** 10.1155/2015/489647

**Published:** 2015-04-27

**Authors:** Mao-Chun Xu, Xiu-Fang Gao, Changwu Ruan, Zhi-Ru Ge, Ji-De Lu, Jian-Jun Zhang, Yu Zhang, Lu Wang, Hai-Ming Shi

**Affiliations:** ^1^Department of Cardiology, Shanghai Pudong New Area Gongli Hospital, Shanghai 200135, China; ^2^Department of Cardiology, Huashan Hospital, Fudan University, Shanghai 200040, China

## Abstract

Oxidative stress plays a critical role in cardiovascular diseases. Salidroside, a glycoside from *Rhodiola rosea*, has been used as an antioxidative therapy for oxidative injury in cardiac diseases. However, the mechanism underlying its antioxidant effect needs to be elucidated. Treatment of HUVECs with H_2_O_2_ significantly decreased the expression of miR-103 in a dose- and time-dependent manner, whereas pretreatment with salidroside significantly inhibited this decrease. Subsequent analysis showed that overexpression of miR-103 abrogated cell activity and ROS production induced by H_2_O_2_. Bcl2/adenovirus E1B 19 kDa interacting protein 3 (BNIP3) was determined to be a novel miR-103 target in HUVECs. Interestingly, H_2_O_2_ treatment upregulated BNIP3 expression; in turn, this effect was inhibited by pretreatment with salidroside. Further studies confirmed that the knockdown of BNIP3 enhanced cell activity and suppressed the ROS production induced by H_2_O_2_. These results demonstrated for the first time that salidroside protects HUVECs in part by upregulating the expression of miR-103, which mediates BNIP3 downregulation and plays an important role in the cytoprotective actions.

## 1. Introduction

Salidroside, an adaptogen purified from* Rhodiola rosea* (*R*.* rosea*), has been reported to exert antioxidative and cardioprotective effects. These findings imply that salidroside may play a central role in the alleviation of mitochondrial-generated ROS and the modulation of mitochondrial-related apoptosis signaling in multiple cell types [1–5]. The mechanisms underlying the antioxidative activity of salidroside have been widely investigated [6]. For example, salidroside was shown to be protective against cardiac dysfunction induced by doxorubicin and against endothelial dysfunction induced by hydrogen peroxide (H_2_O_2_) [7–9]. Functional studies have revealed that salidroside protected neurons of* Caenorhabditis elegans* from polyglutamine-mediated toxicity and SH-SY5Y human neuroblastoma cells against H_2_O_2_-induced cell apoptosis by reducing oxidative stress [1, 10]. Emerging evidence has suggested that specific targeting of microRNAs (miRNAs) by natural agents may open new avenues for the further elucidation of the antioxidative activity of salidroside. To date, very few reports have focused on the effect of salidroside on the expression and function of miRNAs. Determining whether the cytoprotective effects of salidroside are related to the regulation of miRNAs has prompted investigations into cardiovascular pathophysiology.

miRNAs are a class of small endogenous noncoding RNAs that regulate gene expression by interacting with the 3′-UTR of the target mRNAs, thereby indirectly or directly causing their degradation [11–13]. Convincing evidence has demonstrated that miRNAs play critical regulatory roles in diverse biological processes, including metabolism and tumorigenesis [11, 14]. A variety of miRNAs have been shown to be involved in heart development and serve as a diagnostic and prognostic signature [15–17]. miR-103 is a member of the miR-103/107 family located on human chromosome 5 [18]. Recent studies have shown that miR-103 expression was significantly lower in heart failure patients compared with healthy volunteers and therefore could be used as a strong diagnostic predictor [19]. Studies in mouse models have shown that miR-103 protects the mice from hyperphagic obesity by activating the PI3K-Akt-mTOR pathway [20]. Moreover, miR-103 exerts a protective effect against brain stroke damage and neurological deficits by regulating the Na(+)/Ca2+ exchanger in vitro and in vivo [21]. Nevertheless, the mechanism underlying its cardiovascular protective effects remains unclear, especially at the cellular level.

In this study, we investigated changes in miR-103 expression in a cellular model of oxidative stress induced by H_2_O_2_. Then, we assessed the effects of miR-103 on cardiotoxicity in vitro. We demonstrated that miRNA-103 could regulate BNIP3 expression at the translational level. Finally, we revealed that the inhibition of BNIP3 by siRNA rescued cell viability and oxidative damage under a cellular oxidative stress state.

## 2. Materials and Methods

### 2.1. Cell Treatment and Reagents

Human embryonic kidney (HEK293T) cells were obtained from the American Type Culture Collection. Human umbilical vein endothelial cells were conserved in our laboratory and routinely maintained under the culture conditions reported previously [9]. Salidroside was purchased from the National Institute for the Control of Pharmaceutical and Biological Products (Beijing, China). For treatment, cells were cultured with salidroside (100 *μ*g/mL) or a saline vehicle for 24 h, followed by treatment with H_2_O_2_ (200 *μ*M) for 6 h.

### 2.2. Quantitative Real-Time PCR (qRT-PCR) of mRNA and miRNAs

Total RNA was extracted from cultured cells using the TRIzol reagent (Invitrogen, Carlsbad, CA, USA) according to the manufacturer's protocol. For the detection of miR-103 expression, stem-loop reverse transcription-polymerase chain reaction (RT-PCR) was performed using the TaqMan miRNA assay (Applied Biosystems, Foster City, CA, USA). The relative amounts of miR-103 were normalized to the expression of the U6 small RNA.

To measure the mRNA levels of BNIP3, 500 ng of total RNA was reverse transcribed using the Reverse Transcription Kit (Promega, Madison, WI, USA), and qPCR was performed using the TaKaRa SYBR Green PCR Kit (TAKARA, Dalian, China). The relative mRNA expression levels of the BNIP3 gene were normalized to the expression of glyceraldehyde 3-phosphate dehydrogenase (GAPDH). Primers for BNIP3 were designed using Primer Premier 5.0 software (Premier Biosoft International, Palo Alto, CA, USA). The primer BNIP3 sequences were (forward) 5′-TGAGTCTGGACGGAGTAGCTC-3′ and (reverse) 5′-CCCTGTTGGTATCTTGTGGTGT-3′. The primer GAPDH sequences were (forward) 5′-AGCCTTCTCCATGGTGGTGAA-3′ and (reverse) 5′-ATCACCATCTTCCAGGAGCGA-3′. All qRT-PCR reactions were performed with the Applied Biosystems 7900HT Real-Time PCR System (Applied Biosystems, Foster City, CA) in triplicate.

### 2.3. Cell Viability Assay

Each group of cells was plated into 96-well plates at a density of 1 × 10^4^ cells/well. The culture medium was removed after overnight incubation, and then the HUVECs were rinsed with phosphate buffered saline (PBS) and incubated with different treatments. After another 24 h of treatment, cell viability was assessed using the cell counting kit-8 (CCK-8) assay (Doujin Laboratories, Kumamoto, Japan). The absorbance of each well at 450 nm (OD = 450) was read on an ELISA plate reader (Tecan Group Ltd., Männedorf, Switzerland). Three independent experiments were performed in triplicate.

### 2.4. Cell Apoptosis Analysis

Apoptosis was measured by staining with annexin V and propidium iodide using the Annexin V-PE Apoptosis Detection Kit (Abnova, Taipei, Taiwan). Briefly, the cells were treated as indicated, trypsinized, and harvested by centrifugation. Then, the cells were incubated with annexin V and PI for 20 min at room temperature in the dark. The samples were analyzed by flow cytometry using a FACScan cytometer (BD Biosciences, Woburn, MA, USA) and FlowJo software (BD Biosciences).

### 2.5. ROS Determination

HUVECs (10^5^ cells) were seeded into each well of a 24-well plate. The next day, the cells were washed once with PBS and labeled at 37°C for 30 min in culture medium containing 10 *μ*m H2DCFDA (Invitrogen, Inc., Carlsbad, CA, USA). Then, the cells were harvested, washed, and resuspended in PBS and analyzed using a FACScan cytometer (BD Biosciences).

### 2.6. Plasmids Construction and Transient Transfection

To overexpress miR-103, a fragment encoding the pre-miR-103 sequence was amplified by PCR from human genomic DNA (Cwbiotech, Beijing, China) with the following primers: (forward) 5′-AAAGGATCCTACTGCCCTCGGCTTCTTTAC-3′ and (reverse) 5′-AAACTCGAGCAATGCCTTCATAGCCCTGTAC-3′. Then, the PCR product was cloned into the BamHI/XhoI sites of the pCDH-CMV-EF1-copGFP vector (SBI, Mountain View, CA, USA).

To construct the BNIP3 3′-UTR plasmid, a wild-type 3′-UTR fragment of human BNIP3 mRNA containing the putative miR-103 binding sequence was amplified by RT-PCR with the following primers: (forward) 5′-AAACTCGAGTGAAGAACTGGAGTCTGACTTGGTT-3′ and (reverse) 5′-AAAGCGGCCGCCCATTTCCAGTTTTTTAAAGTAGAC-3′. The PCR product was cloned into the XhoI/NotI sites of the psiCHECK-2 vector (Promega, Madison, WI, USA). A mutant of the single miR-103 binding site (5′-AATGCTGC-3′ to 5′-GGGCCTCG-3′) in the 3′-UTR of BNIP3 was generated using the Site-Directed Mutagenesis Kit (Stratagene, San Diego, CA, USA). All plasmids were validated by DNA sequencing.

miRNA mimics, the miRNA inhibitor and the negative control miRNA oligonucleotides for hsa-miR-103, were synthesized by GenePharma (Shanghai, China). A siRNA sequence targeting the human BNIP3 gene was designed and synthesized by GenePharma. The effective siRNA sequence was 5′-GGGCAUAUUCUCUGCAGAAdTdT-3′ (sense). The scrambled siRNA 5′-UUCUCCGAACGUGUCACGUdTdT-3′ (sense) was included as a negative control. HUVECs were transfected with negative control (control) or BNIP3 siRNA at a final concentration of 20 nM using the X-treme GENE siRNA Transfection Reagent (Roche, Indianapolis, IN, USA) according to the manufacturer's instructions. The expression levels of BNIP3 were examined by Western blot analysis 48 h after transfection.

### 2.7. Lentivirus Production

For production of viral particles, lentivirus-mediated miR-103 or the control vector from the pPACKH1 Lentivector Packaging Kit (SBI, Mountain View, CA, USA) was cotransfected into HEK293T cells with the X-tremeGENE siRNA Transfection Reagent. The HUVECs were transduced with miR-103 or the control vector, and the expression of miR-103 was determined by qRT-PCR 72 h after transfection.

### 2.8. Luciferase Assay

For the luciferase reporter assays, HUVECs were seeded into 96-well plates. The cells in each well were transfected with the luciferase reporter plasmid containing either the BNIP3 wild type 3′-UTR (WT) or BNIP3 mutant type 3′-UTR (MUT) sequence and miR-103 mimics or the mimic control using the X-tremeGENE siRNA Transfection Reagent. Luciferase activity was measured 24 h after transfection using a Luciferase Assay Kit (Promega, Madison, WI, USA) following the manufacturer's protocol. The results represent three independent experiments, each performed in triplicate.

### 2.9. Western Blot Analysis

Western blot analysis was performed according to our standard protocol as previously described [9]. Briefly, the cells were lysed with RIPA buffer (CST, Inc., Danvers, MA, USA) containing 1 mM PMSF. Fifty micrograms of total proteins were electrophoresed by SDS-PAGE and the proteins were transferred onto 0.22 *μ*m polyvinylidene difluoride (PVDF) membranes (Millipore, Bedford, MA, USA), followed by blocking with 8% nonfat milk at room temperature for 1 h. The membranes were incubated overnight at 4°C with a rabbit polyclonal anti-BNIP3 antibody (Abnova, Taipei, Taiwan) or anti-GAPDH antibody (Cwbiotech, Shanghai, China), followed by the corresponding horseradish peroxidase- (HRP-) conjugated secondary antibodies (Cwbiotech). The protein bands were visualized using the enhanced chemiluminescence reagents ECL (Millipore, MA, USA), and the signals were densitometrically assessed with the gel analysis tool of the Image J software (National Institute of Health, USA).

### 2.10. Statistical Analysis

Data were expressed as the mean ± standard deviation (SD). The Student's *t*-test and one-way ANOVA test were performed for two groups or multiple groups, respectively. *P* values < 0.05 were considered statistically significant.

## 3. Results

### 3.1. Effects of H_2_O_2_ at Different Concentrations and Time Points on miR-103 Expression

**Figure 1:**
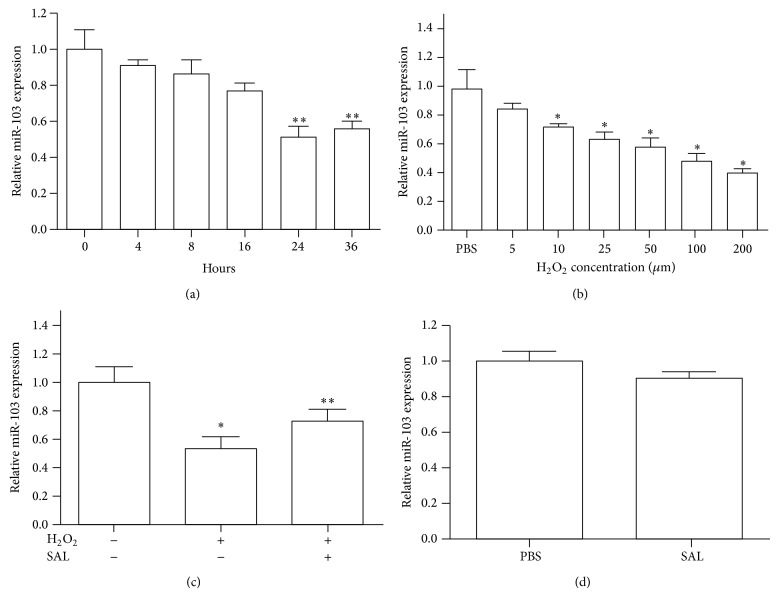
Salidroside mediated the expression of miR-103 in HUVECs induced by H_2_O_2_. (a) The expression of miR-103 was detected by qRT-PCR after H_2_O_2_ treatment at the indicated time points. (b) The expression of miR-103 was determined in HUVECs treated with the indicated concentration of H_2_O_2_ for 6 h. (c) HUVECs were treated with H_2_O_2_ alone or in combination with salidroside. The relative expression of miR-103 was detected by qRT-PCR. (d) qRT-PCR was used to assess the relative expression of miR-103 in HUVECs treated with or without salidroside. Data are shown as the mean ± SD from three independent experiments. ^∗^ *p* < 0.05; ^∗∗^ *p* < 0.01.

A previous report showed that miR-103 played an important role in angiogenesis in vascular endothelial cells [22]. Therefore, we evaluated the expression of miR-103 in HUVECs treated with H_2_O_2_ for different periods of time. As indicated in Figure 1(a), H_2_O_2_ downregulated the expression of miR-103 in a time-dependent manner. To investigate whether H_2_O_2_ mediated the expression of miR-103 in a dose-dependent manner, we treated HUVECs with H_2_O_2_ at concentrations of 5, 10, 25, 50, 100, and 200 *μ*M. H_2_O_2_ decreased the expression of miR-103 when provided at a concentration of 10 *μ*M compared to the control groups (Figure 1(b)). These results provided evidence that H_2_O_2_ reduced the expression of miR-103 in a time- and dose-dependent manner.

### 3.2. Salidroside Attenuates the Inhibition of miR-103 Induced by H_2_O_2_

Our previous study indicated that salidroside protected HUVECs from the cytotoxicity and oxidative stress induced by H_2_O_2_ [9]. To explore whether salidroside augmented the H_2_O_2_-induced inhibition of miR-103 expression, HUVECs were pretreated with salidroside (100 *μ*g/mL) and PBS for 24 h. Using qRT-PCR analysis, we found that the expression trend of miR-103 was reversed in HUVECs induced by H_2_O_2_ induction (Figure 1(c)). Interestingly, we failed to observe a significant increase in miR-103 expression in HUVECs treated with the same concentration of salidroside (100 *μ*g/mL) alone (Figure 1(d)).

### 3.3. Overexpression of miR-103 Inhibited ROS Production and Enhanced Cell Viability in H_2_O_2_-Stimulated HUVECs

**Figure 2:**
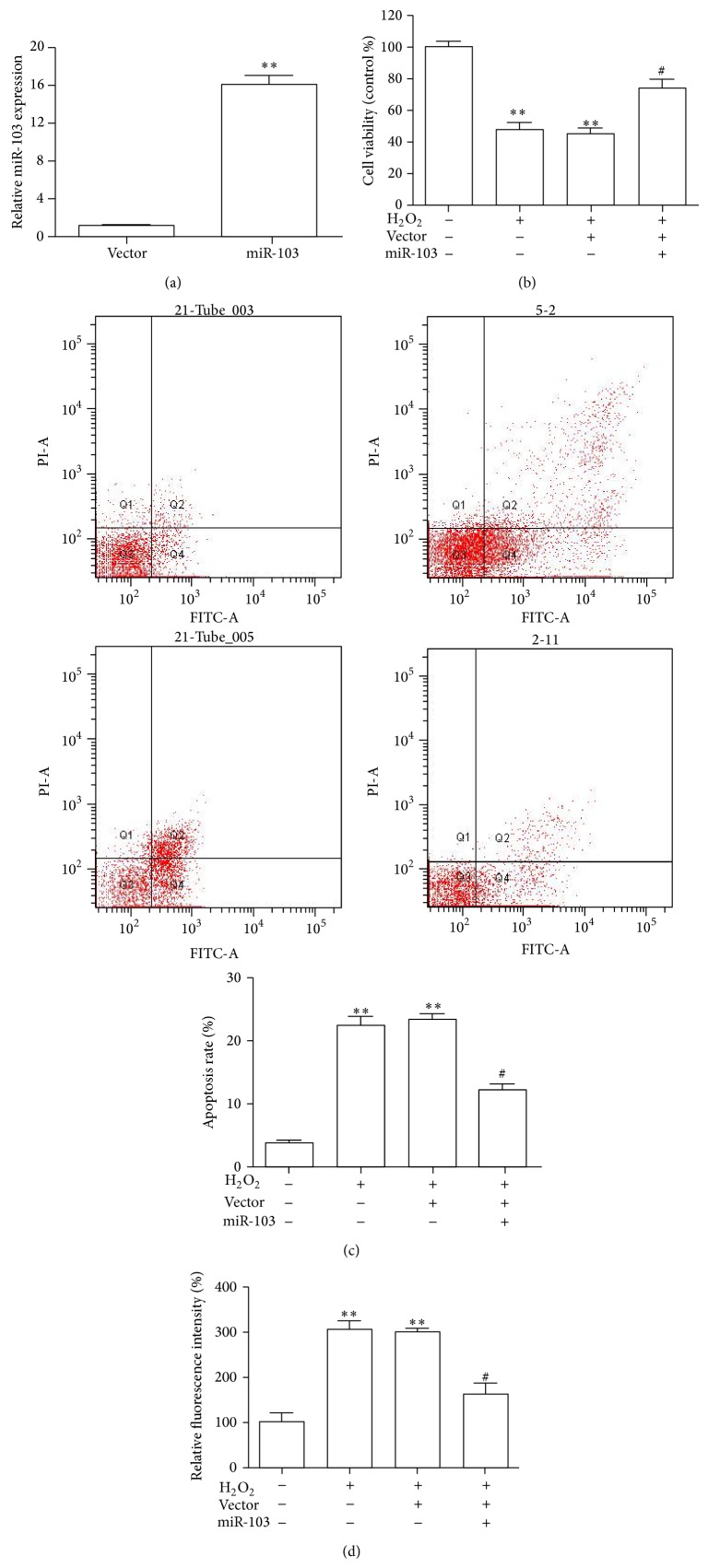
Effect of miR-103 on HUVECs. (a) qRT-PCR was performed to verify the expression of miR-103 in HUVECs transfected with miR-103. (b) Effect of miR-103 on HUVEC viability induced by H_2_O_2_ was measured with the CCK-8 assay. (c) An apoptosis assay was performed to assess the apoptosis levels of HUVECs treated as indicated. (d) Effects of miR-103 on the intracellular formation of ROS triggered by preincubation exposure of HUVECs to H_2_O_2_ assessed by DCF assays. ^∗∗^ *p* < 0.01 versus control and ^#^ *p* < 0.05 versus H_2_O_2_ group.

To assess the biological function of miR-103, HUVECs were stably transduced with miR-103 by lentiviral infection. Empty vector-transfected cells were used as controls. Successful overexpression of miR-103 was confirmed by qRT-PCR (Figure 2(a)). The ectopic overexpression of miR-103 significantly reduced cell viability relative to the control vector (Figure 2(b)). Moreover, apoptosis assays showed that H_2_O_2_ promoted HUVECs apoptosis and necrosis, whereas miR-103 blocked cell apoptosis and necrosis in the transduced cells (Figure 2(c)). Moreover, no significant PBS-related differences were observed in the viabilities of cells treated with or without PBS treatment alone (data not shown). The protective effects of miR-103 against H_2_O_2_-induced oxidative damage were further confirmed by examining their effects on intracellular ROS production (Figure 2(d)). Restoring miR-103 expression significantly decreased the intracellular ROS production induced in HUVECs by H_2_O_2_ treatment. These data indicated that miR-103 was involved in the salidroside-induced attenuation of the damage caused by H_2_O_2_ treatment.

### 3.4. BNIP3 Is Identified as a Target of miR-103

**Figure 3:**
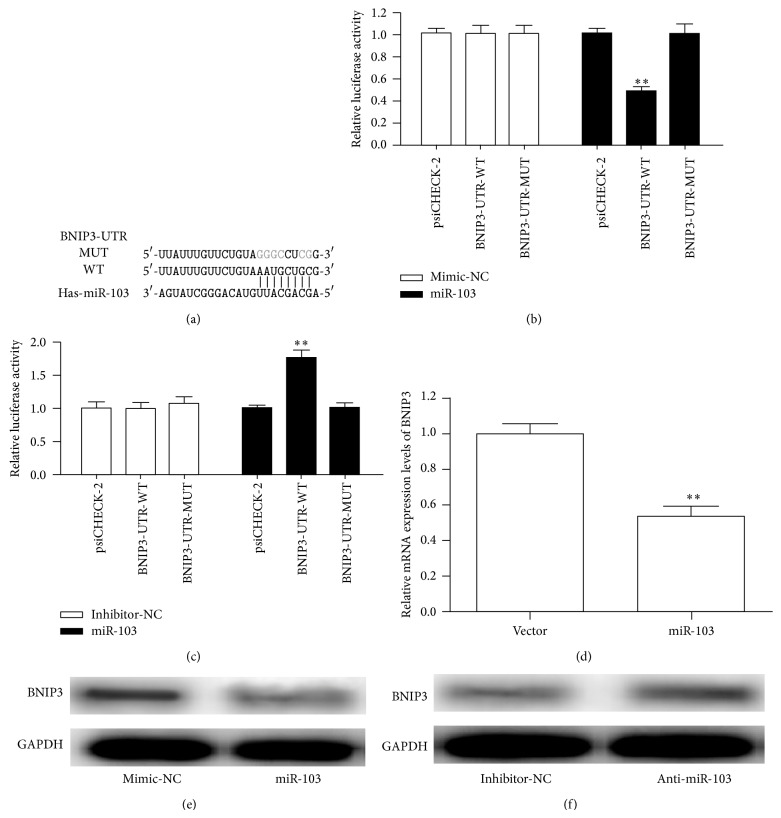
Validation of BNIP3 as a miR-103 target gene. (a) The 3′-UTR of BNIP3 and mutant 3′-UTR sequences that abolished binding. (b) Luciferase activity was assessed in HUVECs transfected with BNIP3 3′-UTR-WT or BNIP3 3′-UTR-MUT and the mimic control or miR-103. (c) Luciferase activity was assessed in HUVECs transfected with BNIP3 3′-UTR-WT or BNIP3 3′-UTR-MUT and the inhibitor control or miR-103 inhibitor. (d) BNIP3 mRNA levels analyzed by qRT-PCR. (e) Western blot analysis of the endogenous expression of BNIP3 upon forced expression of miR-103. (f) The protein expression of BNIP3 in HUVECs transfected with the miR-103 inhibitor or inhibitor control was determined by western blotting. ^∗^ *p* < 0.05; ^∗∗^ *p* < 0.01.

The above findings indicated that miR-103 had a strong protective effect on HUVECs. Next, we searched for potential target genes of miR-103 that might contribute to its function. We performed in silico studies to identify potential gene targets of miR-103 using the bioinformatics algorithms TargetScan (http://www.targetscan.org/) and miRanda (http://www.microrna.org/microrna/home.do). Both algorithms identified BNIP3 as a target of miR-103 based on the putative target sequence of 120–126 bp of the BNIP3 3′-UTR (Figure 3(a)).

Next, we determined whether miR-103 directly targeted the 3′-UTR of BNIP3, thereby potentially contributing to its biological functions. Luciferase reporter plasmids of BNIP3 carrying the 3′-UTR with the potential miR-103 binding site or the miR-103 mutant binding site were cotransfected into HUVECs with either miR-103 or the miRNA negative control. Transfection with miR-103 significantly reduced the luciferase activity compared with the negative control (Figure 3(b)). However, neither miR-103 nor the negative control affected the luciferase activity of the mutant construct. In contrast, inhibition of miR-103 increased the luciferase activity of the BNIP3 3′-UTR compared with the miRNA inhibitor control (Figure 3(c)). Furthermore, cells stably expressing miR-103 showed a clear attenuation of BNIP3 mRNA (Figure 3(d)). To determine whether miR-103 expression could mediate the expression of BNIP3, the BNIP3 protein level was assessed by Western blotting after transfection of miR-103 mimics or miR-103 inhibitors into HUVECs. As demonstrated in Figures 3(e) and 3(f), miR-103 downregulated the expression of BNIP3, while the inhibition of miR-103 upregulated the expression of BNIP3 at the protein level. This finding supported the hypothesis that BNIP3 was a novel direct target of miR-103 in HUVECs.

### 3.5. Salidroside Reduced the Expression of BNIP3 in H_2_O_2_-Stimulated HUVECs

**Figure 4:**
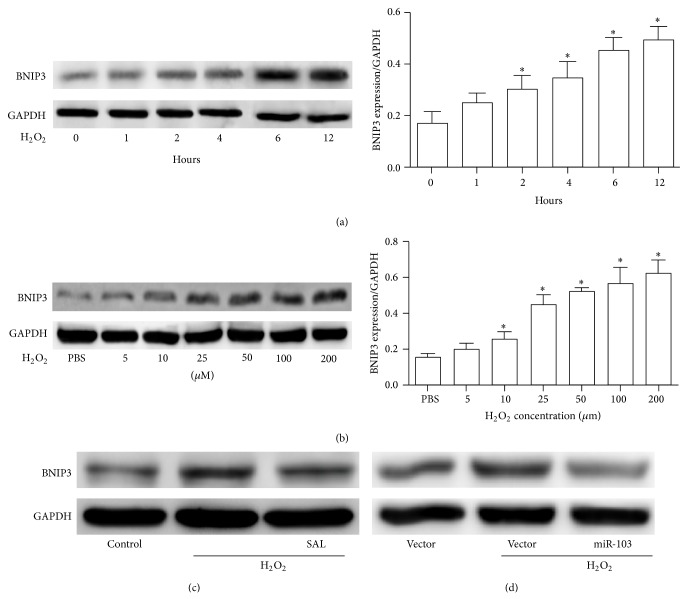
H_2_O_2_ upregulated the expression of BNIP3 in HUVECs. (a) BNIP3 protein expression in HUVECs cultured in 200 *μ*M H_2_O_2_ for different time periods (left). Quantification of BNIP3 by band densitometry analysis (right). (b) BNIP3 protein expression in HUVECs cultured with H_2_O_2_ at the indicated concentration for 6 h (left). Quantification of BNIP3 by band densitometry analysis (right). (c) Western blot analysis of BNIP3 protein expression in HUVECs pretreated with or without salidroside and induced by H_2_O_2_. (d) Western blot analysis of BNIP3 protein expression in HUVECs stably expressing miR-103 treated with H_2_O_2_. GAPDH was used as the loading control. ^∗^ *p* < 0.05; ^∗∗^ *p* < 0.01.

Studies have shown that BNIP3 is a proapoptotic protein that is involved in the pathogenesis of cardiovascular disease through the regulation of mitochondrial functions [23]. Thus, we explored whether H_2_O_2_ regulated the expression of BNIP3 in HUVECs. BNIP3 protein levels were detected by Western blotting after HUVECs were treated with the indicated doses of H_2_O_2_ for the indicated times. As indicated in Figures 4(a) and 4(b), H_2_O_2_ significantly increased the expression of BNIP3 in HUVECs in a time- and concentration-dependent manner (Figures 4(a) and 4(b)). To test whether the upregulation of BNIP3 induced by H_2_O_2_ was inhibited by salidroside in HUVECs, the cells were treated with H_2_O_2_ (200 *μ*M) with or without pretreatment with salidroside for 24 h. BNIP3 was significantly upregulated by pretreatment with salidroside (Figure 4(c)). Similar results were observed when the HUVECs were transfected with miR-103 (Figure 4(d)).

### 3.6. Downregulation of BNIP3 by siRNA Enhanced the Viability and Attenuated ROS Production in HUVECs Treated with H_2_O_2_

**Figure 5:**
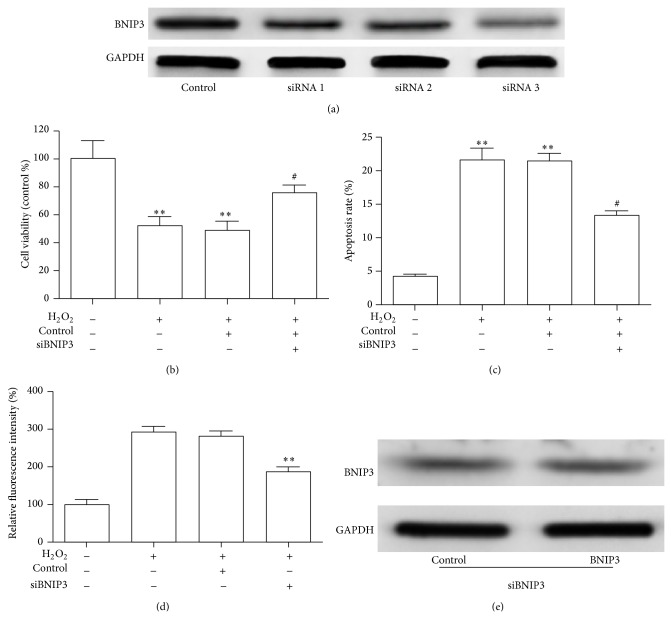
Cell viability and DCF analysis of HUVECs exposed to H_2_O_2_ with or without BNIP3 knockdown. (a) Western blot analysis showing levels of BNIP3 when HUVECs were transfected with different BNIP3 siRNA constructs or a negative control (NC) siRNA. (b) The CCK-8 assay was performed to evaluate the cell viability of HUVECs treated as indicated. (c) An apoptosis assay was used to assess the apoptosis levels of HUVECs treated as indicated. (d) Intracellular formation of ROS was measured in HUVECs treated as indicated. (e) BNIP3 expression was determined in HUVECs transfected with siRNA and treated with or without H_2_O_2_. ^∗^ *p* < 0.05, ^∗∗^ *p* < 0.01 versus control and ^#^ *p* < 0.05 versus H_2_O_2_ group.

Although H_2_O_2_ downregulated the expression of miR-103 and upregulated the expression of BNIP3, whether the downregulation of BNIP3 might play a role in miR-103-induced HUVEC viability and oxidative stress was not clear. Therefore, we inhibited BNIP3 expression in HUVECs treated with H_2_O_2_ using siRNA. BNIP3 protein levels were estimated by Western blotting after transfection of the HUVECs with BNIP3 siRNA 1, siRNA 2, siRNA 3, or the negative control for 48 h. BNIP3 siRNA 3-treated HUVECs showed a significant reduction in BNIP3 compared to the control cells (Figure 5(a)). Based on these results, we chose BNIP3 siRNA 3 (siRNA) as the effective interference sequence for the following experiments. Cell viability was rescued under the H_2_O_2_ treatment in BNIP3 siRNA transfectants (Figure 5(b)). Subsequently, we observed that suppressing BNIP3 expression reversed the cell apoptosis induced by H_2_O_2_ (Figure 5(c)). Furthermore, knockdown of BNIP3 remarkably reduced intracellular ROS production (Figure 5(d)). Although H_2_O_2_ could upregulate the expression of BNIP3, the effects of transfection with siRNA on the downregulation of BNIP3 following treatment with H_2_O_2_ were unknown. Furthermore, the protein levels estimated by Western blotting showed little change after treatment of the cells with H_2_O_2_ (Figure 5(e)). Our findings strongly indicated that salidroside exhibits its protective effects against H_2_O_2_-induced damage of cell viability and oxidative stress by modulating the miR-103/BNIP3 axis.

## 4. Discussion

Oxidative stress is a major stimulus in the pathogenesis of cardiovascular diseases, including atherosclerosis, hypertension, myocardial infarction, and heart failure, via different molecular pathways [9, 24–26]. Hypoxia and ischemia may increase the production of intracellular reactive oxygen species (ROS) that have been demonstrated to inhibit cell growth and induce cell apoptosis and necrosis [27]. Salidroside exhibited unique cardioprotective effects on H9c2 rat cardiomyoblast cells through the activation of the PI3K/Akt pathway and the prevention of the overactivation of oxidative stress-related downstream signaling pathways [7, 28]. Our previous study demonstrated the cardioprotection of salidroside against oxidative stress via the PI3K/Akt/mTOR pathway [9].

Emerging evidence has implicated miRNAs in the regulation of a wide variety of biological processes, such as oxidative stress and apoptosis. However, although miRNAs have emerged as critical players in the cytotoxicity induced by H_2_O_2_, whether the beneficial effects of salidroside were related to miRNAs was unknown. Our results shed some light on the novel role of miR-103 in the oxidative stress of HUVECs.

In the present study, we found that H_2_O_2_ stimulation decreased the expression of miR-103 in a time- and concentration-dependent manner and that salidroside significantly attenuated these changes. However, the expression of miR-103 was not obviously altered in HUVECs. Then, we assessed the cardioprotection function of miR-103 in HUVECs under oxidative stress. Using the CCK-8 assay and flow cytometry, we demonstrated the attenuation of the loss in cell viability and ROS production induced by H_2_O_2_ in HUVECs stably overexpressing miR-103, implying that miR-103 may mimic many features of salidroside in HUVECs treated with H_2_O_2_. Consistent with this notion, experimental studies have revealed that miR-103 affects stroke-induced brain damage and neurological deficits by regulating NCX1 expression [21].

To determine the targets of miR-103 action, in silico algorithms were performed to identify BNIP3 as a potential target of miR-103. BNIP3 is a proapoptotic BH3-only protein that is primarily localized to the mitochondria [29]. BNIP3 has been reported to be upregulated in the heart after acute ischemia and in chronic heart failure patients after myocardial infarction [30, 31]. Additionally, reports have suggested that hypoxia-activated cardiac myocyte death due to increasing BNIP3 expression might be associated with mitochondrial dysfunction [32–34]. Our results demonstrated that miR-103 directly targeted the 3′-UTR of BNIP3 because its overexpression was associated with the suppression of luciferase activity. Moreover, the overexpression of miR-103 caused a significant downregulation of endogenous BNIP3 RNA and protein levels, indicating that BNIP3 gene transcripts are direct targets of miR-103. To further investigate whether salidroside affected the expression of BNIP3, we treated H_2_O_2_-induced HUVECs with salidroside or transfection with miR-103 mimics. The results showed that both treatment with salidroside and treatment with transfection with miR-103 mimics downregulated BNIP3 expression. However, whether BNIP3 is involved in the oxidative stress was not completely delineated. The use of siRNAs against BNIP3 in HUVECs could effectively inhibit the expression of BNIP3. Furthermore, we found that siRNA-mediated inhibition of BNIP3 resulted in reduced levels of ROS production and improved cell viability.

## 5. Conclusions

In this report, we demonstrated that miR-103 was upregulated following pretreatment with salidroside in an H_2_O_2_-induced oxidative stress model of HUVECs, thereby dramatically decreasing the level of BNIP3 mRNA and protein expression by directly binding to the 3′-UTR of BNIP3. Thus, modulation of miR-103 levels may provide a new strategic therapeutic target for oxidative stress-associated disorders. However, the molecular mechanism by which miR-103 protects cultured HUVECs against oxidative stress needs to be further elucidated.
